# Clinical significance of circulating tumor cells and tumor markers in the diagnosis of lung cancer

**DOI:** 10.1002/cam4.2286

**Published:** 2019-05-27

**Authors:** Yang Li, Xudong Tian, Lei Gao, Xiaohong Jiang, Rao Fu, Tingting Zhang, Tianying Ren, Ping Hu, Yaping Wu, Peige Zhao, Dawei Yang

**Affiliations:** ^1^ Zhong Yuan Academy of Biological Medicine Liaocheng People's Hospital Liaocheng China; ^2^ Department of Thoracic Surgery Liaocheng People's Hospital Liaocheng China; ^3^ Department of Respiratory Medicine Liaocheng People's Hospital Liaocheng China

**Keywords:** circulating tumor cells, lung cancer, NE‐FISH, tumor markers

## Abstract

**Background:**

Lung cancer has the highest fatality rate of all cancer types. To improve patients’ survival and life quality, it is therefore very important to screen for and detect it at an early stage.

**Methods:**

A negative enrichment–fluorescence in situ hybridization (NE‐FISH) approach was used to detect circulating tumor cells (CTCs) in lung cancer patients, and levels of lung cancer‐associated serum markers were also measured in the peripheral blood of these same patients. The correlation between CTCs, serum cancer markers (carcinoembryonic antigen [CEA], CA 125, CYFRA 21‐1, and SCC), and clinicopathological characteristics was then investigated. Moreover, the potential clinical use of the combination of CTCs and tumor markers for the diagnosis of lung cancer, especially at early stages, was also explored.

**Results:**

CTC frequencies in lung cancer patients were significantly higher than in healthy control volunteers or patients with benign lung disease, and the area under the receiver operating characteristics curve for the control group was 0.846 (95% CI 0.796‐0.887, *P* < 0.001). The rate of CTC positivity in lung cancer patients was 68.29% when the CTC cutoff value was 2, and the sensitivity of this means of lung cancer detection rose to 82.93% by combining CTC‐based detection with measurements of serum tumor markers. Similarly, the diagnostic sensitivity of this approach in early‐stage lung cancer patients (I‐II) was improved from 63.93% to 78.69%. Detection of CTCs can thus assist with the identification of benign and malignant pulmonary nodules.

**Conclusions:**

It is potentially helpful and effective to employ a combination of CTCs and serum tumor markers for the clinical diagnosis of lung cancer.

## 1 INTRODUCTION

Cancer incidence and mortality are constantly increasing, and as such cancer remains a major public health problem worldwide.^1^ Lung cancer has the highest fatality rate of all cancers both in China and globally.^2^ Although the diagnosis, treatment, and care of lung cancer patients have all improved in recent years, most lung cancer patients have a poor prognosis with an overall 5‐year survival rate of 18.1% because they are in advanced stages of disease when first diagnosed.^3^ Early detection of lung cancer is thus important to improve overall survival. It is therefore necessary to identify biomarkers which can be used to diagnose lung cancer at an early stage and to monitor the dissemination of tumor cells within the body.

Currently, imaging‐based screening, tumor markers, and histopathological methods are the primary approaches used to diagnose lung cancer. However, tumor lesions are usually small at an early stage, limiting the sensitivity of imaging techniques for detection. Histopathology, the gold standard for tumor diagnosis at the moment, cannot be utilized as a means of dynamic real‐time monitoring because of the associated trauma to the body. In addition, although serum levels of tumor markers such as carcinoembryonic antigen (CEA), fragments of cytokeratin‐19 (CYFRA21‐1), and neuron‐specific enolase (NSE) are commonly measured for the diagnosis of lung cancer, the results are often not very specific or reliable, with false‐positive results often occurring due to infections, benign tumors, pregnancy, or other factors.^4^

Circulating tumor cells (CTCs) refer to the cancer cells that have escaped from the primary tumor and disseminated into the bloodstream or lymphatic system. They can spread to other organs and give rise to metastatic tumors.^5^ Previous research has shown that CTCs can also cause tumor recurrence and are related to patient prognosis.^6^, ^7^ The ability to obtain more information from CTCs may therefore offer an avenue toward the early detection of cancer, allowing researchers to gain insight into its the aggressive nature of the tumor and offering a means of monitoring therapeutic responses and disease progression in patients.^8^, ^9^, ^10^

In 2004, the CellSearch system was approved by FDA as a means of detecting CTCs in breast cancer, prostate cancer, and colorectal cancer patients given their promise as biomarkers useful for monitoring chemotherapeutic efficacy.^11^, ^12^, ^13^ These CellSearch systems relied upon detecting CTCs based on their surface expression of epithelial cell adhesion molecules (EpCAM) and cytokeratins (CK).^14^, ^15^ However, expression of EpCAM and CK is very dynamic on different types or stages of cancer cells, especially those undergoing the epithelial‐to‐mesenchymal transition process, leading to a lower CTC detection rate and restricting the clinical application of this strategy as a means of detecting CTCs.^16^, ^17^ Therefore, developing an assay not reliant upon EpCAM for effective capture and identification of additional subtypes of CTCs is imperative. Because chromosomal instability can cause aneuploidy in human solid tumor cells,^18^, ^19^ an assay integrating EpCAM‐independent subtraction and immunostaining‐fluorescence in situ hybridization (FISH) has been reported in previous studies.^9^, ^20^, ^21^, ^22^, ^23^, ^24^, ^25^, ^26^

In this study, an EpCAM‐independent enrichment strategy and FISH were used to detect CTCs in lung cancer patients.^27^ The relationship between CTC numbers, the levels of particular tumor markers (CEA, CA 125, CYFRA 21‐1, and SCC), and the clinicopathological factors in these patients were also analyzed. Moreover, the potential clinical use of the combination of CTCs and tumor markers for the diagnosis of lung cancer was explored as well.

## 2 MATERIALS AND METHODS

### 2.1 Patients and specimens

The current study included 174 patients with lung cancer and 90 control individuals at Liaocheng People's Hospital (Liaocheng, Shandong, China) who were newly diagnosed without any prior treatment between June 2017 and October 2018. All cancer patients, including 14 cases of small cell lung cancer (SCLC) and 160 of none‐small cell lung cancer (NSCLC), (113 adenocarcinomas and 47 squamous cell carcinomas), were confirmed by histopathological diagnosis. Thirty‐seven patients with benign lung diseases and 53 healthy donors in the control group were age and gender matched to the cancer patients, with no statistically significant differences between these groups (Table S1). Patients with benign diseases were diagnosed by imaging, fiber optic bronchoscopy, and histopathology. All patients provided written informed consent to participate in this study. This study was approved by Liaocheng People's Hospital and was conducted in accordance with the principles of the Declaration of Helsinki.

Peripheral blood samples (3.2 mL) were collected from each patient into a Vacutainer tube (BD, Franklin, NJ), then kept at room temperature for CTC detection. In addition, serum samples used for tumor marker detection were obtained via venous puncture and collected in anticoagulant‐free blood‐collecting tubes on the same day. The measurement of tumor markers, including CEA, CA125, CYFRA 21‐1, NSE, SCC, and Pro‐GRP, was not inclusion criteria and was not mandatory for enrollment in this study. To avoid bias, the collection, encoding, and detection of all blood samples were performed in a blinded manner by different personnel.

### 2.2 Enrichment and identification of CTCs

**Figure 1:**
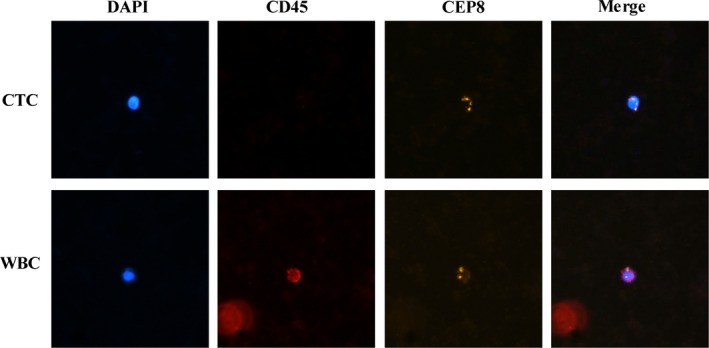
Identification of CTCs by NE‐FISH. CEP, centromere probe; CTCs, circulating tumor cells; NE‐FISH, negative enrichment‐fluorescence in situ hybridization; WBC, white blood cells

CTC detection was performed via a negative enrichment‐fluorescence in situ hybridization (NE‐FISH) method as previously described.^27^ A microscope (BX63, Olympus) was then used to scan the slides, and image analyses were performed using an automated image analysis system—the IMSTAR high content screening (HCS) device (IMSTAR SA, France). CTCs were identified as DAPI+/CD45−/CEP 8+ cells (Figure 1).

### 2.3 CTC detection stability test

The human A549 lung cancer cell line was obtained from the Shanghai Institute for Biological Sciences and cultured in F‐12K medium with 10% fetal bovine serum at 37°C in 5% CO_2_. Before the test, Mito‐Tracker Green was used to label the cancer cells. 1, 5, 10, 20, or 40 of labeled cells were then added to 3.2 mL of healthy donor blood. The tumor cells were then enriched and identified via the NE‐FISH method. The recovery rate was calculated as the ratio of recovered cell numbers after enrichment to the number of spiked‐in cells. Result identification was performed by experienced technicians or the IMSTAR HCS device.

### 2.4 Measurement of tumor markers

Serum tumor markers, including CEA, CA 125, CYFRA 21‐1, NSE, SCC, and Pro‐GRP, were analyzed using an immunology analyzer (Cobas e602; Roche Diagnostics, Germany). We considered 5 ng/mL, 35 U/mL, 3.3 ng/mL, 35 ng/mL, 1.5 ng/mL, and 63 pg/mL as the upper limits of normality for CEA, CA 125, CYFRA 21‐1, NSE, SCC, and Pro‐GRP, respectively.

### 2.5 Statistical analysis

Statistical analyses were performed using SPSS (version 17, SPSS Inc, Chicago, IL). A receiver operating characteristics curve (ROC) was used to determine the cutoff value for the number of CTCs used to diagnose lung cancer. The differences in the areas under curve (AUC_ROC_) were calculated using MedCalc 18.2.1. Graphs were generated using GraphPad Prism 5 (GrapPad Software, La Jolla, CA, USA). *P* values were calculated using two‐sided tests and were considered statistically significant at the *P* < 0.05 level.

## 3 RESULTS

### 3.1 CTC detection stability test

The recovery rate of A549 lung cancer cells spiked into healthy blood samples enriched by our negative enrichment strategy was greater than 80%, as shown in Table 1. The detection rate using the IMSTAR HCS device was higher than that achieved by experienced technicians. Although most of the cells in the peripheral blood were leukocytes, the majority of these were removed using immunomagnetic anti‐CD45 beads via negative enrichment. In order to determine the total cell numbers and cell types in each sample, we randomly selected one hundred slides and counted the number of cells present thereupon, determining that 9943 ± 7384 (mean ± SD) DAPI‐positive cells and 311 ± 162 CD45‐negative cells were present on each slide (Table S2). The rate of CD45‐negative cells on these slides was only 4.16 ± 2.22%, which was quite low, and almost 95% of these CD45‐negative cells were CD45 false‐negative cells, as their low CD45 expression could not be discerned by IMSTAR. It is ultimately easier to identify the true‐negative cells manually, at a time cost of 5‐10 minutes per slide. Therefore, using the IMSTAR HCS device, we can count cells on four slides within 1 hour, whereas experienced technicians usually require 1 hour per slide, making the IMSTAR HCS device more stable, objective, repeatable, and efficient than counting manually.

**Table 1:** CTC detection stability test

Spiked cell number	Recovered	Recovery rate (%)^a^	Detected			
			Technologist	IMSTAR HCS device^b^		
				Cycle 1	Cycle 2	Cycle 3
1	1	100	1	1	1	1
5	4	80	4	4	4	4
10	8	80	7	8	8	7
20	17	85	15	16	17	17
40	33	82.5	31	33	32	33

Abbreviations: CTC, circulating tumor cell; HCS, high content screening.

a Recovery rate = recovered cell number/spiked cell number.

b The number of CTCs on each slide was read out by IMSTAR HCS device three times.

### 3.2 Detection of CTCs in cancer patients and controls

**Figure 2:**
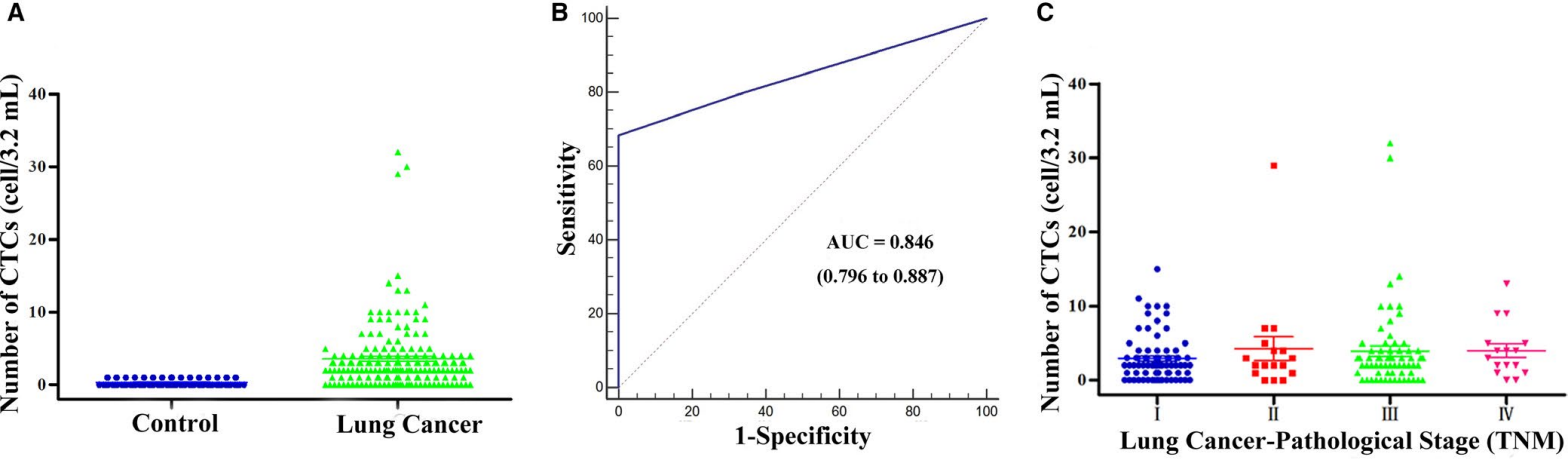
CTC counts in patients and controls. A, Distribution of CTCs in controls and lung cancer. B, ROC curves used for determine the cutoff value for CTCs. C, Distribution of CTCs in patients with different pathological stage. CTCs, circulating tumor cells; ROC, receiver operating characteristics curve

We are able to detect CTCs in 3.2 mL of blood in the control group in 35.85% of healthy donors with a median of 0 cells/samples (range: 0‐1), and in 27.03% of patients with benign disease with a median of 0 cells/samples (range: 0‐1). We are also able to detect CTCs in 138 cases (79.31%) from the 174 lung cancer patients in this study, with a median of 2 cells/samples (range: 0‐32). A significant difference in the rate of positive CTC detection was identified between lung cancer patients and the control group (*P* < 0.001, Figure 2A). Receiver operator characteristic curve (ROC) was used to distinguish between lung cancer patients and control group and to assess the sensitivity and specificity of CTCs as a diagnostic tool, revealing these values to be 68.39% and 100% (Figure 2B), respectively. CTCs could be used for the diagnosis of lung cancer when the cutoff value was 1.5 CTCs/3.2 mL of blood (AUC = 0.846, 95% CI 0.796‐0.887, *P* < 0.001). Cutoff values of 1 CTC and 2 CTCs yielded respective sensitivities of 79.31% and 68.39%, and specificities of 67.78% and 100%, respectively. Therefore, we elected to use a 2 CTC cutoff value for the diagnosis of lung cancer.

**Figure 3:**
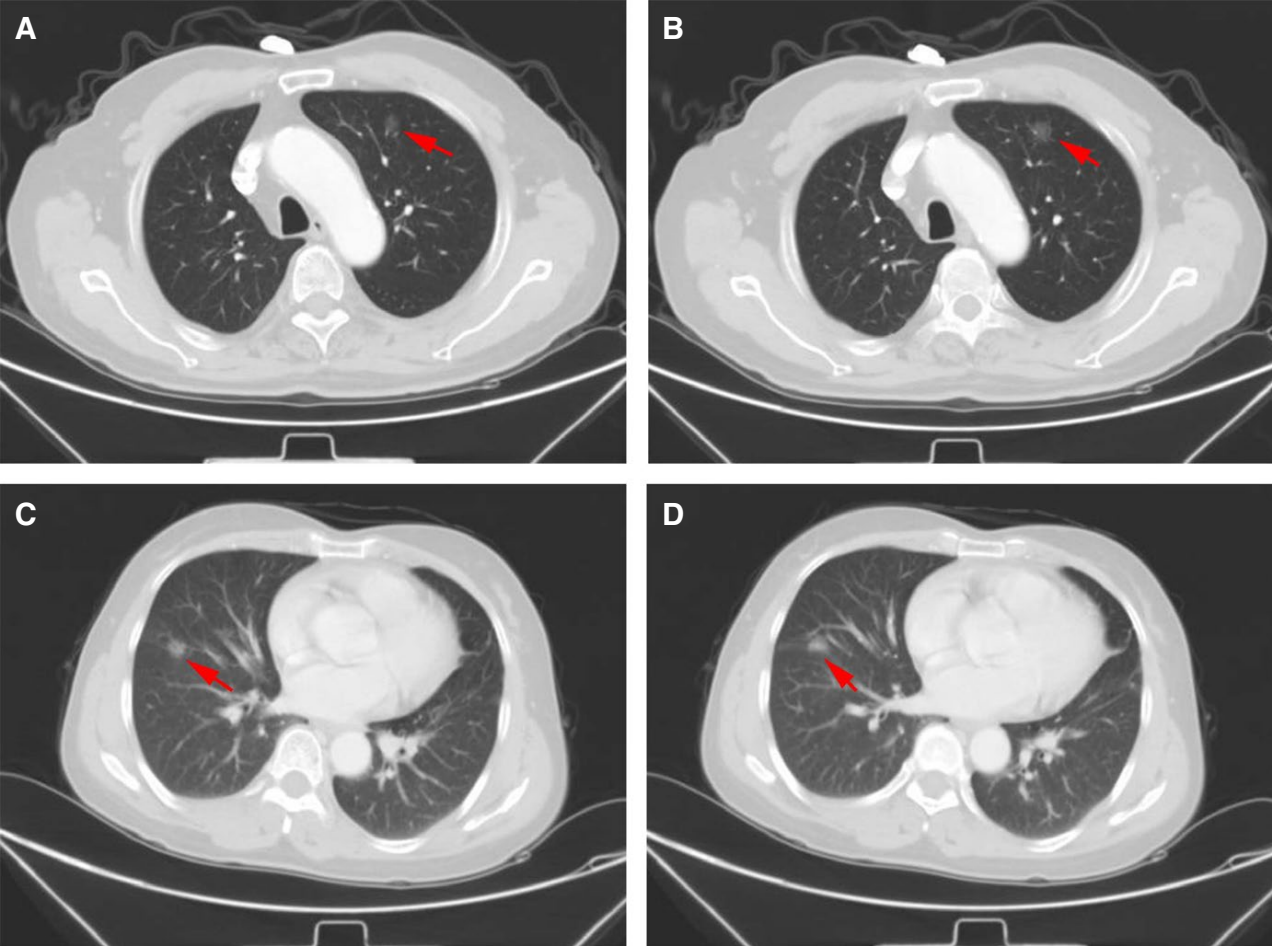
CT scans of the lung cancer patients. A, B, 1.3‐cm ground glass nodule was present in the left upper lobe of the lung. C, D, 1.0‐cm ground glass nodule was present in the right middle lobe of the lung

Detection of CTCs can assist with the discrimination between benign and malignant pulmonary nodules. For example, in one case, a 68‐year‐old man underwent chest radiography (CT) while being evaluated for a cough, and a 1.3‐cm ground glass nodule is discovered (Figure 3A,B). For this patient, 6 CTCs were detected by NE‐FISH; however, serum cancer markers including CEA, CA 125, CYFRA 21‐1, NSE, SCC, and Pro‐GRP were normal. After surgical resection, the pathological diagnosis of the resultant biopsy was early‐stage lung adenocarcinoma (IA). In another case, a 48‐year‐old woman was hospitalized for right pulmonary space occupying, and a 1.0‐cm ground glass nodule was found in her right lung upon by CT examination (Figure 3C,D). Our approach detected 5 CTCs in this patient, and yet all serum cancer markers were normal. The postoperative pathologic diagnosis of this patient was also early‐stage lung adenocarcinoma (IA). These results were consistent with the use of a 2 CTC cutoff value for the diagnosis of lung cancer, revealing this approach to have better diagnostic efficacy than conventional serum cancer markers.

### 3.3 The relationship between CTCs and clinicopathological characteristics

The number of detected CTCs ranged from 0 to 29 CTCs per 3.2 mL blood sample (median: 2) and 0‐32 CTCs per 3.2mL blood sample (median: 3) in patients with stage I‐II or stage III‐IV cancer, respectively (*P* = 0.287; Figure 2C). Two or more CTCs were detected in 68.39% of patients with lung cancer. The relationship between CTC positivity and clinicopathological characteristics is shown in Table 2. Patients with NSCLC and SCLC had CTC positivity rates (≥2 per 3.2 mL) of 69.38% and 57.14%, respectively. For those with NSCLC, the CTC‐positive rates were 68.14% for adenocarcinomas and 72.34% for squamous cell carcinomas. No significant differences were observed in clinicopathological characteristics as a function of different CTC counts. In those patients with adenocarcinomas, we found that CTC counts differed significantly with tumor depth (*P* = 0.001, Table S3). In the present study, the observed 68.39% rate of CTC positivity among lung cancer patients was slightly lower than in previous studies.^28^, ^29^ This maybe because more early‐stage I patients were enrolled in this study, or it may be a consequence of intra‐tumor heterogeneity.

**Table 2:** Relationship of CTC with patient demographics and clinical characteristics

Characteristics	n	Proportion (%)	CTC <2		CTC ≥2		*P*
			n	Proportion (%)	n	Proportion (%)	
Gender							
Male	112	64.37	29	25.89	83	74.11	0.311
Female	62	35.63	26	41.94	36	58.06	
Age							
≥60	122	70.11	36	29.51	86	70.49	0.530
<60	52	29.89	19	36.54	33	63.46	
Smoking history							
Yes	85	48.85	25	29.41	60	70.59	0.134
No	89	51.15	30	33.71	59	66.29	
Histology							
Adenocarcinoma	113	64.94	36	31.86	77	68.14	0.557
Squamous	47	27.01	13	27.66	34	72.34	
SCLC	14	8.05	6	42.86	8	57.14	
Distant metastasis							
M0	159	91.38	51	32.08	108	67.92	0.512
M1	15	8.62	4	26.67	11	73.33	
Tumor depth							
T1	88	50.57	31	35.23	57	64.77	0.059
T2	60	34.48	20	33.33	40	66.67	
T3	17	9.77	1	5.88	16	94.12	
T4	9	5.17	3	33.33	6	66.67	
Lymph node metastasis							
Yes	92	52.87	26	28.26	66	71.74	0.911
No	82	47.13	29	35.37	53	64.63	
TNM stage (UIUC)							
I	74	42.53	26	35.14	48	64.86	0.872
II	17	9.77	5	29.41	12	70.59	
III	67	38.51	20	29.85	47	70.15	
IV	16	9.20	4	25.00	12	75.00	

Abbreviation: CTC, circulating tumor cell; TNM, tumor‐node‐metastasis.

### 3.4 Serum tumor markers in cancer patients

As measurements of tumor markers were not an inclusion criterion for this study and were not mandatory for enrollment, only 123 of the lung cancer patients and 59 control donors had available serum tumor marker data.

**Figure 4:**
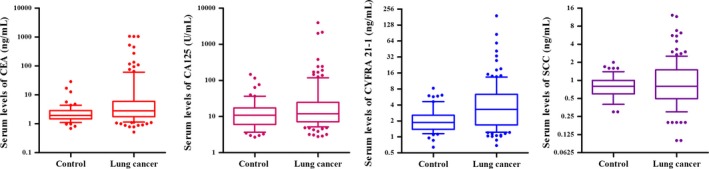
Serum levels of CEA, CA 125, CYFRA 21‐1, and SCC in control and lung cancer (box plot with median, 10, 25, 75, and 90 centiles). CEA, carcinoembryonic antigen

The median age of these patients was 65 years (range: 36‐83 years), and the majority of them were male (64.23%), had adenocarcinomas (60.98%), had lymph node metastasis (56.91%), and had no distant metastases (89.43%) in the lung cancer group. Serum tumor marker sensitivity was 28.46% (35 patients) for CEA, 19.51% (24 patients) for CA 125, 3.25% (4 patients) for NSE, 50.41% (62 patients) for CYFRA 21‐1, 26.02% (32 patients) for SCC, and 11.38% (14 patients) for Pro‐GRP. Because of the small number of patients who presented with abnormal levels of NSE and Pro‐GRP, we did not take these two markers into account for further analysis. Serum levels of tumor markers, including CEA, CA 125, CYFRA 21‐1, and SCC, are shown in Figure 4.

### 3.5 Correlation of tumor markers with clinicopathological characteristics

Table 3 demonstrates the relationship between tumor markers, patient demographics, and clinical characteristics. The median levels of CEA, CA 125, CYFRA 21‐1, and SCC were 2.80 ng/mL, 12.1 U/mL, 3.31 ng/mL, 0.80 ng/mL, respectively. Patients with a history of smoking presented with significantly higher levels of CEA (*P* = 0.043) and CA 125 (*P* = 0.025) than did nonsmoking individuals. A statistically significant correlation was observed between SCC levels of different histological classifications (*P* < 0.001) and tumor depth (*P* = 0.040). We also found that the levels of CEA, CA 125, and CYFRA 21‐1 differed significantly based on whether patients had distant metastases (*P* = 0.049), based on gender (*P* = 0.023), and based on whether patients had lymph node metastases (*P* = 0.022), respectively.

**Table 3:** Relationship of tumor markers (CEA, CA 125, CYFRA 21‐1, and SCC) with patient demographics and clinical characteristics

Characteristics	n	CEA		*P*	CA 125		*P*	CYFRA 21‐1		*P*	SCC		*P*
		>5 ng/mL, %	Median (IQR)		>35 U/mL, %	Median (IQR)		>3.3 ng/mL, %	Median (IQR)		≥1.5 ng/mL, %	Median (IQR)	
Gender													
Male	79	35.44	3.25 (4.89)	0.607	17.72	12.80 (18.30)	0.023	60.76	3.89 (5.00)	0.562	35.44	1.00 (1.20)	0.830
Female	44	15.91	2.20 (2.61)		22.73	11.75 (14.05)		31.82	2.27 (2.70)		9.09	0.60 (0.50)	
Age													
≥60	83	28.92	2.86 (3.64)	0.834	21.69	12.80 (17.80)	0.859	54.22	3.43 (4.87)	0.925	27.71	0.80 (1.00)	0.127
<60	40	27.50	2.19 (5.30)		15.00	10.15 (16.40)		42.50	2.74 (2.84)		22.50	0.80 (0.98)	
Smoking history													
Yes	62	33.87	3.49 (4.05)	0.043	22.58	16.10 (22.30)	0.025	67.74	4.21 (4.86)	0.653	30.65	0.90 (1.23)	0.280
No	61	22.95	2.18 (3.29)		16.39	10.40 (12.75)		32.79	2.29 (2.48)		21.31	0.70 (0.65)	
Histology													
Adenocarcinoma	75	32.00	2.84 (7.69)	0.193	22.67	11.40 (19.00)	0.598	42.67	2.46 (3.07)	0.148	12.00	0.70 (0.50)	0.000
Squamous	35	22.86	2.79 (2.88)		11.43	13.30 (13.80)		77.14	4.82 (3.62)		60.00	1.70 (1.30)	
SCLC	13	23.08	2.18 (3.04)		23.08	11.80 (37.15)		23.08	1.81 (3.68)		15.38	0.70 (0.70)	
Distant metastasis													
M0	110	24.55	2.65 (3.03)	0.049	16.36	11.55 (15.83)	0.698	48.18	3.28 (4.59)	0.508	27.27	0.90 (1.03)	0.950
M1	13	61.54	9.09 (189.71)		46.15	19.10 (197.45)		69.23	4.50 (6.50)		15.38	0.60 (0.60)	
Tumor depth													
T1	59	22.03	2.35 (2.75)	0.629	8.47	9.20 (10.20)	0.347	28.81	2.00 (1.95)	0.316	22.03	0.80 (0.70)	0.040
T2	49	34.69	3.44 (16.54)		32.65	17.40 (58.05)		63.27	4.16 (4.32)		24.49	0.70 (1.05)	
T3	10	40.00	3.13 (4.28)		30.00	22.10 (36.93)		90.00	5.54 (2.81)		40.00	1.05 (0.98)	
T4	5	20.00	2.27 (3.35)		0.00	21.60 (14.25)		100.00	7.25 (31.37)		60.00	1.60 (0.80)	
Lymph node metastasis													
Yes	70	45.71	4.25 (16.81)	0.515	32.86	20.95 (62.93)	0.772	68.57	5.54 (4.60)	0.022	34.29	0.90 (1.20)	0.317
No	53	5.66	2.09 (1.87)		1.89	8.10 (8.35)		26.42	2.01 (1.95)		15.09	0.70 (0.50)	
TNM stage (UIUC)													
I	50	6.00	2.14 (1.91)	0.453	0.00	8.00 (6.55)	0.666	22.00	2.00 (1.75)	0.168	16.00	0.65 (0.50)	0.132
II	11	27.27	2.79 (10.07)		18.18	14.30 (20.20)		81.82	6.83 (10.47)		45.45	1.10 (2.50)	
III	48	41.47	3.40 (8.85)		33.33	21.85 (65.30)		68.75	5.26 (4.40)		35.42	0.95 (1.05)	
IV	14	64.29	7.53 (147.79)		42.86	16.50 (178.48)		64.29	4.20 (5.80)		14.29	0.55 (0.60)	

Abbreviations: CEA, carcinoembryonic antigen; CTC, circulating tumor cell.

CEA, as a glycoprotein involved in cell adhesion, varies significantly across histological lung cancer types, with the most elevated serum levels of CEA being associated with adenocarcinomas.^30^ CA 125 is another tumor marker produced by adenocarcinomas and useful in the differential diagnosis of adenocarcinomas.^31^ Table 4 illustrates the relationship of CEA and CA 125 levels with patient demographics and clinical characteristics in those with adenocarcinomas. The median levels of CEA and CA 125 were 2.84 ng/mL and 11.40 U/mL in these patients. No significant differences were observed in clinicopathological characteristics as a function of CEA levels; however, CA 125 levels differed significantly as a function of tumor depth (*P* = 0.020), lymph node metastasis (*P* = 0.027), and TNM stage (*P* = 0.007).

**Table 4:** Relationship of tumor markers (CEA and CA 125) with patient demographics and clinical characteristics in adenocarcinoma cancer

Characteristics	n	CEA		*P*	CA 125		*P*
		>5 ng/mL, %	Median (IQR)		>35 U/mL, %	Median (IQR)	
Gender							
Male	39	43.59	3.25 (20.74)	0.650	23.08	13.90 (20.90)	0.492
Female	36	19.44	2.38 (2.86)		22.22	11.20 (14.70)	
Age							
≥60	49	34.69	3.23 (12.24)	0.925	22.45	12.50 (19.60)	0.857
<60	26	26.92	2.28 (7.75)		23.08	10.10 (24.45)	
Smoking history							
Yes	28	12	3.42 (21.95)	0.400	32.14	22.25 (94.63)	0.209
No	47	12	2.35 (5.43)		17.02	9.40 (13.40)	
Distant metastasis							
M0	65	26.15	2.61 (3.87)	0.182	16.92	10.10 (16.10)	0.247
M1	10	70.00	40.84 (317.37)		60.00	86.85 (229.15)	
Tumor depth							
T1	46	21.74	2.19 (2.83)	0.297	10.87	8.70 (8.75)	0.020
T2	24	50.00	4.95 (98.11)		41.67	21.95 (110.85)	
T3	4	50.00	4.43 (70.74)		50.00	31.30 (57.75)	
T4	1	0.00	3.23 (0.00)		0.00	21.6 (0)	
Lymph node metastasis							
Yes	33	66.67	11.07 (110.30)	0.659	48.48	24.70 (140.45)	0.027
No	42	4.76	2.10 (1.87)		2.38	7.70 (6.35)	
TNM stage (UIUC)							
I	41	4.88	2.11 (1.88)	0.418	0.00	7.50 (6.15)	0.007
II	3	66.67	45.87 (133.02)		66.67	70.80 (86.30)	
III	20	60.00	8.25 (84.45)		45.00	24.60 (113.53)	
IV	11	72.73	26.64 (272.59)		54.55	47.70 (229.80)	

Abbreviation: CEA, carcinoembryonic antigen.

CYFRA 21‐1 is a sensitive tumor marker for NSCLC, with particular sensitivity for squamous cell lung cancer.^32^, ^33^ In our study, the sensitivity of CYFRA 21‐1 was 77.14%, making it the most sensitive tumor markers for squamous cancer. SCC was the second most sensitive tumor marker for squamous cancer, with the sensitivity of 60.00%. Even so, no significant differences were found in clinicopathological characteristics as a function of CYFRA 21‐1 or SSC levels (Table S4).

### 3.6 Combination of CTCs and tumor markers for the diagnosis of lung cancer

**Figure 5:**
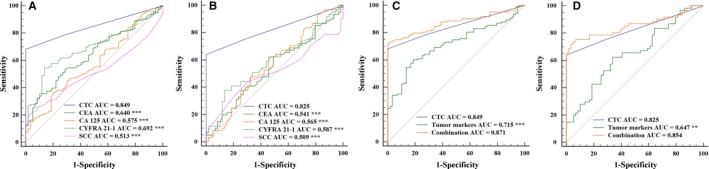
Comparison between ROC curves in different diagnostic methods. A, Comparison of CTC, CEA, CA 125, CYFRA 21‐1, and SCC detection in 123 of lung cancer patients and 59 donors in control group. B, Comparison of CTC, CEA, CA 125, CYFRA 21‐1, and SCC detection in 61 of lung cancer patients with early stage and 59 donors in control group. C, Comparison among CTC, tumor markers (CEA, CA 125, CYFRA 21‐1, and SCC), and their combination detection in 123 of lung cancer patients and 59 donors in control group. D, Comparison among CTC, tumor markers (CEA, CA 125, CYFRA 21‐1, and SCC), and their combination detection in 61 of lung cancer patients with early stage and 59 donors in control group. ***P < 0.001; **P < 0.01. CEA, carcinoembryonic antigen; CTC, circulating tumor cell

A total of 123 of lung cancer patients and 59 control donors underwent simultaneous CTC and tumor marker detection, and these patients were used to compare the diagnostic performance of those markers. The diagnostic efficacy of CTC detection in the peripheral blood (AUC_ROC_ = 0.849) was significantly higher than that of CEA, CA 125, CYFRA 21‐1, and SCC (AUC_ROC_ = 0.640, 0.575, 0.692, and 0.513, respectively, all *P* < 0.001, Figure 5A). The same findings were also evident in patients with early‐stage cancer (I‐II, AUC_ROC_ = 0.825 vs 0.541, 0.565, 0.587, and 0.509, respectively, all *P* < 0.001, Figure 5B).

We also explored the combination of CTC and tumor markers for the diagnosis of lung cancer. We found that the sensitivity of CTCs and a combination of tumor markers (CEA, CA 125, CYFRA 21‐1, and SCC) for lung cancer diagnosis to be 68.29% (AUC_ROC_ = 0.849) and 63.41% (AUC_ROC_ = 0.715), respectively. However, the combination of all of those markers for the diagnosis of lung cancer was more accurate, with a sensitivity of 82.93% (AUC_ROC_ = 0.871, Figure 5C). For patients with early‐stage disease (I‐II), the sensitivity was 78.69% (AUC_ROC_ = 0.854 vs 0.825, 0.647, Figure 5D) using this combination of all tested markers.

The combination of CTCs and tumor markers for the diagnosis of adenocarcinomas and squamous cell cancer were also compared, revealing the same improvements for each of these two histological classifications. This approach was particularly beneficial for diagnosing squamous cell cancer, with a sensitivity of 97.14% using the combination of CTCs, CYFRA 21‐1, and SCC (AUC_ROC_ = 0.960 vs 0.892, 0.898, Figure S1A), which lends support to the potential use of the combined analysis of CTCs, CYFRA 21‐1, and SCC as a complementary tool for the diagnosis of squamous cell cancer. Using a combination of CTCs, CEA, and CA 125, the diagnosis of adenocarcinomas was also more accurate, with a sensitivity of 77.33% (AUC_ROC_ = 0.874 vs 0.845, 0.647, Figure S1B). For adenocarcinoma patients with early‐stage disease, the sensitivity of using this combination was not improved as compared to CTCs alone (AUC_ROC_ = 0.856 vs 0.819, 0.648, Figure S1C).

## 4 DISCUSSION

In this study, we used NE‐FISH to detect CTCs in lung cancer patients. In this way, a relatively limited amount of peripheral blood (3.2 mL) was used for CTC analysis as compared to the 7.5 mL peripheral blood used in previous CTC analyses.^15^, ^22^, ^23^, ^34^ A stability test of our CTC detection approach indicated that this strategy had a high detection sensitivity, with the IMSTAR HCS device being a more stable, objective, repeatable, and efficient approach to CTC quantification than counting manually. Using a cutoff value of 2 CTCs, the sensitivity and specificity rates of using CTC number to identify patients with lung cancer were 68.39% and 100%, respectively. We did not detect any relationship between CTC counts and the clinicopathological characteristics of lung cancer. There is a clear need for further confirmation of the association between CTC counts and clinicopathological parameters in a larger patient cohort. We did, however, find that for patients with adenocarcinomas, CTC counts differed significantly as a function of tumor depth (*P* = 0.001).

Pulmonary nodules may appear as either solid or subsolid masses which do not completely obscure adjacent tissues. Eighty percent of pulmonary nodules are solid, while 20% are subsolid.^35^ A subsolid pulmonary nodule can be further subclassified as either a pure ground glass nodule or a partial solid nodule. It is very difficult to determine whether a pure ground glass nodule is benign or malignant based upon CT examination alone. The detection of CTCs cannot replace CT imaging or core biopsy for diagnostic patients with suspicious malignant lung lesions.^36^ However, detection of CTCs has the potential to provide more information about pulmonary nodules and is of value in helping clinicians to decide on the appropriate treatment in pulmonary nodules.

Tumor markers have been extensively used in the clinic for diagnosing lung cancer, and for predicting patient prognosis. Even so, these markers can be detected in some benign lesions, leading to high false‐negative/positive rates.^37^, ^38^ Most publications report that a combination of several tumor markers provides high sensitivity, but the most useful combination of such markers remains unclear.^39^, ^40^, ^41^ In our study, the combination of four tumor markers—CEA, CA 125, CYFRA 21‐1, and SCC—yielded had a lung cancer diagnostic sensitivity of 63.41%, which was lower than that achieved based on CTC counts alone.

Moreover, the combination of CTC counts and tumor marker levels for the diagnosis of lung cancer was also explored. Using both of these cancer indicators, the diagnostic sensitivity of this approach for lung cancer detection was bolstered to 82.93%, and for squamous cancer, this positive diagnosis rate, using the combination of CTCs, CYFRA 21‐1, and SCC, was 97.14%. In addition, 78.69% of patients in the early stages (I and II) of disease could be diagnosed using a combination of CTCs, CEA, CA 125, CYFRA 21‐1, and SCC, and such an approach has excellent potential as a means of facilitating early cancer diagnosis that will influence patient treatment decisions in order to allow for the better management of lung cancer in the clinic.

## 5 CONCLUSIONS

A new platform, including EpCAM‐independent enrichment strategy, FISH, and IMSTAR HCS device, was used for the detection of CTCs in patients with lung cancer in this study. Using a cutoff value of 2 CTCs in 3.2 mL of blood, the sensitivity and specificity of this approach for the diagnosis of lung cancer were 68.39% and 100%, respectively, indicating that the analysis of peripheral blood CTCs using this platform has a clear potential value for the diagnosis of lung cancer. Additionally, the diagnostic rate was improved when a combination of CTCs and tumor markers was used to identify patients affected by lung cancer. Although further research is needed to verify our results, these findings have provided some reference for the diagnosis of lung cancer.

## CONFLICT OF INTEREST

The authors declare no conflict of interest.

## Data Availability

The data can be provided by the corresponding author when upon requests.
