# Supplementary material for: Clinical significance of circulating tumor cells and tumor markers in the diagnosis of lung cancer
Source: Cancer Med. 2019 May 27;8(8):3782–92. doi: 10.1002/cam4.2286 (PMC6639255; doi:10.1002/cam4.2286)
Supplement: Supplementary file 1 [file CAM4-8-3782-s001.docx]

**Clinical significance of circulating tumor cells and tumor markers in diagnosis of lung cancer**

**TABLE S1** Comparison of basic characteristics between control and cancer group

|  | **Lung cancer** | | ***P*** |
| --- | --- | --- | --- |
|  | **Control** | **Test** |  |
| Gender |  |  |  |
| Male (%) | 68.89 | 64.37 | 0.464 |
| Female (%) | 31.11 | 35.63 |  |
| Age (y), mean ± SD | 65.2 ± 13.1 | 63.0 ± 8.6 |  |
| ≥60 (%) | 72.22 | 70.11 | 0.110 |
| <60 (%) | 27.78 | 29.89 |  |

**TABLE S2** The cell staining of each slide

| **No.** | **Number of DAPI active cells** | **Number of CD45 negative cells** | **Rate of CD45 negative cells (%)** | **No.** | **Number of DAPI active cells** | **Number of CD45 negative cells** | **Rate of CD45 negative cells (%)** | **No.** | **Number of DAPI active cells** | **Number of CD45 negative cells** | **Rate of CD45 negative cells (%)** | **No.** | **Number of DAPI active cells** | **Number of CD45 negative cells** | **Rate of CD45 negative cells (%)** |
| --- | --- | --- | --- | --- | --- | --- | --- | --- | --- | --- | --- | --- | --- | --- | --- |
| 1 | 14239 | 802 | 5.6 | 27 | 32462 | 383 | 1.2 | 53 | 8037 | 356 | 4.4 | 79 | 4722 | 320 | 6.8 |
| 2 | 8386 | 507 | 6.0 | 28 | 28605 | 216 | 0.8 | 54 | 6760 | 436 | 6.4 | 80 | 8510 | 271 | 3.2 |
| 3 | 6400 | 380 | 5.9 | 29 | 5382 | 172 | 3.2 | 55 | 1760 | 183 | 10.4 | 81 | 2542 | 145 | 5.7 |
| 4 | 9170 | 216 | 2.4 | 30 | 8035 | 146 | 1.8 | 56 | 2358 | 160 | 6.8 | 82 | 12209 | 320 | 2.6 |
| 5 | 5217 | 241 | 4.6 | 31 | 8321 | 592 | 7.1 | 57 | 6523 | 342 | 5.2 | 83 | 4355 | 138 | 3.2 |
| 6 | 1906 | 128 | 6.7 | 32 | 25780 | 353 | 1.4 | 58 | 5262 | 309 | 5.9 | 84 | 3387 | 253 | 7.5 |
| 7 | 15927 | 807 | 5.1 | 33 | 1125 | 11 | 1.0 | 59 | 12660 | 223 | 1.8 | 85 | 22800 | 483 | 2.1 |
| 8 | 6207 | 359 | 5.8 | 34 | 5435 | 62 | 1.1 | 60 | 998 | 56 | 5.6 | 86 | 7603 | 308 | 4.1 |
| 9 | 26316 | 225 | 0.9 | 35 | 16618 | 633 | 3.8 | 61 | 3829 | 218 | 5.7 | 87 | 4811 | 222 | 4.6 |
| 10 | 9225 | 235 | 2.5 | 36 | 2581 | 129 | 4.6 | 62 | 2407 | 152 | 6.3 | 88 | 2710 | 118 | 4.4 |
| 11 | 10841 | 209 | 1.9 | 37 | 12207 | 490 | 4.0 | 63 | 12980 | 395 | 3.0 | 89 | 8353 | 455 | 5.4 |
| 12 | 9395 | 552 | 5.9 | 38 | 9607 | 357 | 3.7 | 64 | 6598 | 487 | 7.4 | 90 | 3102 | 97 | 3.1 |
| 13 | 15886 | 651 | 4.1 | 39 | 12035 | 425 | 3.5 | 65 | 3838 | 287 | 7.5 | 91 | 7675 | 356 | 4.6 |
| 14 | 11733 | 391 | 3.3 | 40 | 14394 | 335 | 2.3 | 66 | 13371 | 547 | 4.1 | 92 | 10325 | 525 | 5.1 |
| 15 | 20947 | 437 | 2.1 | 41 | 12188 | 298 | 2.4 | 67 | 1093 | 121 | 11.1 | 93 | 9045 | 514 | 5.7 |
| 16 | 24781 | 309 | 1.3 | 42 | 9008 | 384 | 4.3 | 68 | 1537 | 175 | 11.4 | 94 | 11224 | 391 | 3.5 |
| 17 | 6274 | 182 | 2.9 | 43 | 5002 | 180 | 3.6 | 69 | 9782 | 236 | 2.4 | 95 | 4904 | 353 | 7.2 |
| 18 | 6053 | 224 | 3.7 | 44 | 9162 | 397 | 4.3 | 70 | 7101 | 373 | 5.3 | 96 | 8190 | 185 | 2.3 |
| 19 | 2825 | 90 | 3.2 | 45 | 8485 | 168 | 2.0 | 71 | 17871 | 562 | 3.1 | 97 | 15997 | 750 | 4.7 |
| 20 | 3311 | 162 | 4.9 | 46 | 13422 | 280 | 2.1 | 72 | 4652 | 198 | 4.3 | 98 | 7405 | 280 | 2.8 |
| 21 | 2394 | 175 | 7.3 | 47 | 12739 | 419 | 3.3 | 73 | 6024 | 264 | 4.4 | 99 | 3986 | 172 | 4.3 |
| 22 | 22586 | 444 | 2.0 | 48 | 4379 | 225 | 5.1 | 74 | 3178 | 59 | 1.9 | 100 | 7878 | 247 | 3.1 |
| 23 | 16880 | 329 | 1.9 | 49 | 21825 | 193 | 0.9 | 75 | 3173 | 280 | 8.8 | mean ± SD | 9,943±7384 | 311±162 | 4.16±2.22 |
| 24 | 26512 | 158 | 0.6 | 50 | 32579 | 291 | 0.9 | 76 | 17479 | 459 | 2.6 |  |  |  |  |
| 25 | 12960 | 469 | 3.6 | 51 | 3720 | 233 | 6.3 | 77 | 5630 | 264 | 4.7 |  |  |  |  |
| 26 | 26860 | 411 | 1.5 | 52 | 4681 | 240 | 5.1 | 78 | 14653 | 339 | 2.3 |  |  |  |  |

**TABLE S3** Relationship of CTC with patient demographics and clinical characteristics in adenocarcinoma cancer

| **Characteristics** | ***n*** | **Proportion (%)** | **CTC <2** | | **CTC ≥2** | | ***P*** |
| --- | --- | --- | --- | --- | --- | --- | --- |
|  |  |  | ***n*** | **Proportion (%)** | ***n*** | **Proportion (%)** |  |
| Gender |  |  |  |  |  |  |  |
| Male | 59 | 52.21 | 15 | 25.42 | 44 | 74.58 | 0.377 |
| Female | 54 | 47.79 | 21 | 38.89 | 33 | 61.11 |  |
| Age |  |  |  |  |  |  |  |
| ≥60 | 75 | 66.37 | 23 | 30.67 | 52 | 69.33 | 0.978 |
| <60 | 38 | 33.63 | 13 | 34.21 | 25 | 65.79 |  |
| Smoking History |  |  |  |  |  |  |  |
| Yes | 41 | 36.28 | 12 | 29.27 | 29 | 70.73 | 0.236 |
| No | 72 | 63.72 | 24 | 33.33 | 48 | 66.67 |  |
| Distant Metastasis |  |  |  |  |  |  |  |
| M0 | 101 | 89.38 | 32 | 31.68 | 69 | 68.32 | 0.308 |
| M1 | 12 | 10.62 | 4 | 33.33 | 8 | 66.67 |  |
| Tumor depth |  |  |  |  |  |  |  |
| T1 | 73 | 64.60 | 23 | 31.51 | 50 | 68.49 | 0.001 |
| T2 | 29 | 25.66 | 13 | 44.83 | 16 | 55.17 |  |
| T3 | 8 | 7.08 | 0 | 0.00 | 8 | 100.00 |  |
| T4 | 3 | 2.65 | 0 | 0.00 | 3 | 100.00 |  |
| Lymph node metastasis |  |  |  |  |  |  |  |
| Yes | 48 | 42.48 | 14 | 29.17 | 34 | 70.83 | 0.875 |
| No | 65 | 57.52 | 22 | 33.85 | 43 | 66.15 |  |
| TNM stage (UIUC) |  |  |  |  |  |  |  |
| I | 61 | 53.98 | 21 | 34.43 | 40 | 65.57 | 0.396 |
| II | 6 | 5.31 | 2 | 33.33 | 4 | 66.67 |  |
| III | 33 | 29.20 | 9 | 27.27 | 24 | 72.73 |  |
| IV | 13 | 11.50 | 4 | 30.77 | 9 | 69.23 |  |

**TABLE S4** Relationship of tumor markers (CYFRA 21-1 and SCC) with patient demographics and clinical characteristics in squamous cell lung cancer

| **Characteristics** | ***n*** | **CYFRA 21-1** | | ***P*** | **SCC** | | ***P*** |
| --- | --- | --- | --- | --- | --- | --- | --- |
|  |  | **>3.3 ng/mL, %** | **Median (IQR)** |  | **≥1.5 ng/mL, %** | **Median (IQR)** |  |
| Gender |  |  |  |  |  |  |  |
| Male | 31 | 83.87 | 5.49 (3.82) | 0.609 | 61.29 | 1.70 (1.80) | 0.838 |
| Female | 4 | 25.00 | 3.01 (2.62) |  | 50.00 | 1.35 (0.70) |  |
| Age |  |  |  |  |  |  |  |
| ≥60 | 25 | 84.00 | 5.71 (3.72) | 0.827 | 64.00 | 1.90 (1.90) | 0.808 |
| <60 | 10 | 60.00 | 4.41 (5.91) |  | 50.00 | 1.50 (1.35) |  |
| Smoking History |  |  |  |  |  |  |  |
| Yes | 26 | 80.77 | 5.16 (3.57) | 0.366 | 50.00 | 1.50 (1.43) | 0.151 |
| No | 9 | 66.67 | 3.77 (11.68) |  | 88.89 | 1.70 (2.70) |  |
| Distant Metastasis |  |  |  |  |  |  |  |
| M0 | 34 | 76.47 | 4.73 (3.71) | 0.889 | 58.82 | 1.70 (1.45) | 0.631 |
| M1 | 1 | 100.00 | 6.50 (0.00) |  | 100.00 | 1.60 (0.00) |  |
| Tumor depth |  |  |  |  |  |  |  |
| T1 | 11 | 54.55 | 3.37 (4.22) | 0.509 | 54.55 | 1.70 (1.50) | 0.641 |
| T2 | 15 | 80.00 | 6.31 (3.56) |  | 66.67 | 2.10 (2.30) |  |
| T3 | 5 | 100.00 | 4.24 (2.03) |  | 60.00 | 1.50 (1.25) |  |
| T4 | 4 | 100.00 | 10.77 (42.78) |  | 50.00 | 1.30 (0.83) |  |
| Lymph node metastasis |  |  |  |  |  |  |  |
| Yes | 24 | 91.97 | 6.83 (9.52) | 0.301 | 66.67 | 1.75 (1.95) | 0.864 |
| No | 11 | 45.45 | 3.27 (1.15) |  | 45.45 | 1.00 (1.50) |  |
| TNM stage (UIUC) |  |  |  |  |  |  |  |
| I | 9 | 33.33 | 3.06 (0.88) | 0.760 | 55.56 | 1.70 (1.55) | 0.572 |
| II | 8 | 87.50 | 6.91 (13.12) |  | 62.50 | 2.45 (9.08) |  |
| III | 17 | 94.12 | 5.71 (6.74) |  | 58.82 | 1.60 (1.50) |  |
| IV | 1 | 100.00 | 6.50 (0.00) |  | 100.00 | 1.60 (0.00) |  |

**
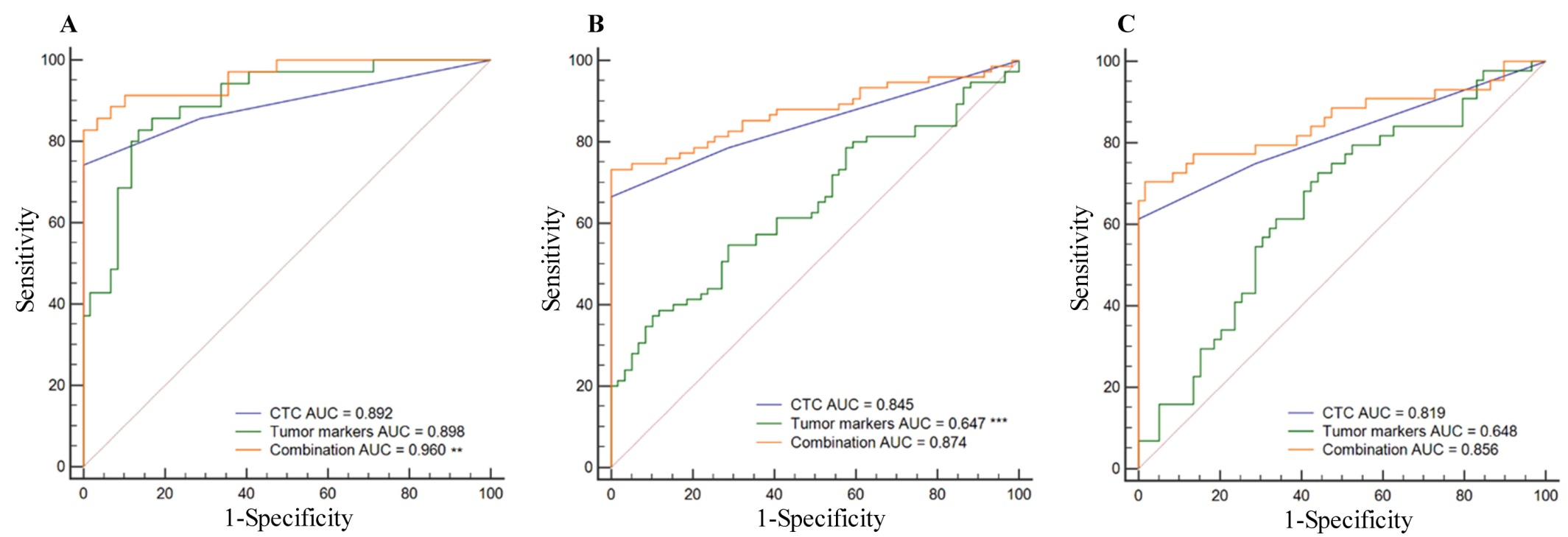
**

**FIGURE S1** Comparison between ROC curves in different diagnostic methods. A, Comparison among CTC, tumor markers (CYFRA 21-1 and SCC), and their combination detection in 35 of squamous cell cancer patients and 59 donors in control group. B, Comparison among CTC, tumor markers (CEA and CA 125), and their combination detection in 75 of adenocarcinomas patients and 59 donors in control group. C, Comparison among CTC, tumor markers (CEA and CA 125), and their combination detection in 44 of adenocarcinomas patients with early stage and 59 donors in control group. *** *P* < 0.001; ** *P* < 0.01; CTC, circulating tumor cells; ROC, receiver operator characteristic; AUC, areas under curves.
